# High expression of lncRNA PELATON serves as a risk factor for the incidence and prognosis of acute coronary syndrome

**DOI:** 10.1038/s41598-022-11260-2

**Published:** 2022-05-16

**Authors:** Linmu Chen, Yunxiu Huang

**Affiliations:** 1 Department of Pharmacy, Sun Yat-sen University Affiliated Zhongshan Hospital, Zhongshan, 528403 Guangdong Province China; 2 Department of Laboratory Medicine, Sun Yat-sen University Affiliated Zhongshan Hospital, Zhongshan, 528403 Guangdong Province China

**Keywords:** Diagnostic markers, Predictive markers, Prognostic markers

## Abstract

Atherosclerosis is the primary origin of acute coronary syndrome (ACS) diseases. Previous studies have shown that lncRNA plaque-enriched long noncoding RNA in atherosclerotic macrophage regulation (lncRNA PELATON) is a specific lncRNA in macrophage nuclei. This study aims to identify serum lncRNA PELATON as a biomarker for assessing the incidence and prognosis of ACS. Levels of serum lncRNA PELATON were detected by real-time polymerase chain reaction (RT–PCR) in patients with ACS and healthy individuals. The clinical significance of lncRNA PELATON in patients with ACS was assessed by analyzing receiver operating characteristic and survival curves. The serum levels of lncRNA PELATON in patients with ACS were significantly higher than those in healthy individuals. LncRNA PELATON expression was positively correlated with the expression levels of high sensitivity C-reactive protein (hs-CRP), cardiac troponin T (cTnT) and creatine kinase MB (CK-MB) (*p* < 0.05). LncRNA PELATON can be used as a potential diagnostic index with an AUC of 0.706 for unstable angina pectoris (UA), 0.782 for acute non-ST-segment elevation myocardial infarction (NSTEMI) and 0.900 for acute ST-segment elevation myocardial infarction (STEMI). The incidence of major cardiovascular events in patients with ACS with high lncRNA PELATON expression was higher than that in those with low lncRNA PELATON expression. However, the mortality between patients in the high and low lncRNA PELATON groups was not significantly different. This study showed that higher levels of lncRNA PELATON were negatively correlated with the prognosis of ACS, revealing the potential of this measurement to serve as an index to assess the incidence and prognosis of ACS.

## Introduction

China is one of the countries with the heaviest burden of cardiovascular diseases in the world^1^. In China, cardiovascular diseases are the main cause of death^2,3^, accounting for 40% of population deaths^4^. Acute coronary syndrome (ACS) is a group of clinical syndromes, including acute ST-segment elevation myocardial infarction (STEMI), acute non-ST-segment elevation myocardial infarction (NSTEMI), and unstable angina pectoris (UA), based on the rupture or invasion of coronary atherosclerotic plaque^5^. Coronary angiography is a common and effective method for the diagnosis of coronary atherosclerotic heart disease^6^. It is a relatively safe and reliable invasive diagnostic technique that has been widely used in clinical practice, and it is considered as the "gold standard" for the diagnosis of coronary heart disease^7^. However, some patients, such as those who are allergic to iodine or contrast agents, have severe cardiopulmonary insufficiency, have uncontrolled severe arrhythmias, have electrolyte disorders, and have severe hepatic and renal insufficiency, cannot undergo invasive operations. Moreover, complications, such as coronary artery perforation, cardiac tamponade, embolism of vital organs, and cervical and mediastinal hematoma limit its use^8^. Therefore, it is particularly important to find new and effective early diagnostic indicators for acute coronary syndrome.

LncRNA, which is a class of RNA molecules with sequences that lack an open reading frame, is a functional molecule existing in various species^9^. It has obvious cell specificity and can fold to form many second-order structures with active energy, and its recording process is regulated by dynamic states^10^. Based on publicly available lncRNA-related databases, novel lncRNA-disease associations have been predicted by multiple computational prediction models, such as the Improved Random Walk with Restart for LncRNA-Disease Association prediction (IRWRLDA)^11^ and the KATZ measure for LncRNA-Disease Association prediction (KATZLDA)^12^. Combining computational prediction models with experimental validation, lncRNAs are believed to be closely associated with a variety of diseases in the human body, such as cancer, heart failure and Alzheimer's disease^12–15^. In the state of cardiovascular disease, many lncRNAs are differentially expressed^16,17^.

LncRNA ANRIL has been identified as an important risk factor for coronary artery disease because of its involvement in the regulation of histone methylation^18,19^. In addition, lncRNA MI-related transcripts were associated with myocardial infarction (MI)^20^. Seven candidate lncRNA biomarkers were identified by analyzing the lncRNA expression profiles of 52 ACS patients. In addition, functional analysis of GO and KEGG suggested that these seven lncRNAs may be involved in MI-related biological processes and pathways^21^. Further studies found that lncRNAs were better than miRNAs or mRNA expression profiles for predicting heart failure in different pathological states, suggesting that lncRNAs have important pathophysiological functions in the cardiovascular system^22^.

LncRNA PELATON, located on chromosome 20q13.13 and containing 3 exon regions with a total length of 539 nt, is a specific lncRNA in the macrophage nucleus^23^. Previously, it was also known as RP11-290F20.3, GCRL1 (gastric cancer-related lncRNA 1) and SMIM-25 (small membrane integral protein 25)^24^. Comparing atherosclerotic carotid arteries from unstable patients to those with stable disease, LncRNA PELATON was identified as being the most significantly upregulated lncRNA among a collection of other transcripts differentially expressed in plaques^25^. Hung et al. found that it was specifically expressed in monocyte-macrophage cell lines and could not be detected in endothelial cells, smooth muscle cells or fibroblasts^23^. The lncRNA PELATON can enhance the phagocytic capacity of macrophages and increase the production of reactive oxygen species by increasing CD36 expression on the macrophage membrane^23^, but the mechanism has not been further explored. As a chronic disease with a large accumulation of macrophage foam cells, atherosclerosis is the primary origin of acute coronary syndrome diseases (ACS). Considering the important role of monocytes, macrophages and foam cells in the formation of atherosclerosis, we speculated that lncRNA PELATON might be an important factor for AS disease.

Thus, the clinical relevance of lncRNA PELATON for the diagnosis and prognosis of ACS was explored through RT–PCR, receiver operating characteristic (ROC) curve and survival analysis to further optimize the individualized treatment of the disease and improve the survival rate of ACS patients.

## Results

### Clinical characteristics of the study participants

The clinical characteristics of 98 ACS individuals and 37 healthy individuals participating in this study are shown in Table 1.

**Table 1:** Characteristics of the ACS individuals and healthy individuals.

	Healthy individuals (n = 37)	ACS individuals (n = 98)	χ2/t	p
Age (years)	48 ± 8	50 ± 13		0.287
Gender (man/femal)	15/22	44/54	0.207	0.648
BMI	23.24 ± 3.43	24.05 ± 3.83		0.264
Hypertension (Y/N)	10/27	63/35	15.01	0.0001
Diabetes mellitus (Y/N)	7/30	36/62	3.927	0.04
WBC (10^9^/L)	6.59 ± 0.80	6.97 ± 1.08		0.06
Neu%	61.29 ± 6.60	62.94 ± 11.05		0.396
AST (U/L)	23.43 ± 9.51	24.46 ± 11.75		0.162
LDH (U/L)	176.48 ± 46.99	191.78 ± 71.15		0.229
CK (U/L)	127.68 ± 24.80	141.31 ± 49.74		0.114
α-HBDH (U/L)	133.49 ± 33.69	162.76 ± 33.07		0.000
mAST (U/L)	9.05 ± 4.97	9.41 ± 3.72		0.652
TG (mmol/L)	1.36 ± 0.34	2.03 ± 0.64		0.000
TC (mmol/L)	4.58 ± 0.80	5.50 ± 0.91		0.000
LDL-C (mmol/L)	2.85 ± 0.61	3.51 ± 0.56		0.000
HDL-C (mmol/L)	1.12 ± 0.23	1.11 ± 0.28		0.000
Lp(a) (mg/L)	163.89 ± 26.77	540.92 ± 112.19		0.000
ApoA1 (g/L)	1.06 ± 0.24	1.96 ± 0.48		0.000
ApoB100 (g/L)	0.94 ± 0.13	1.45 ± 0.53		0.000
sLDL-C (mmol/L)	0.37 ± 0.07	1.51 ± 0.42		0.000
NEFA (mmol/L)	0.16 ± 0.03	0.74 ± 0.22		0.000
Hcy (μmol/L)	5.18 ± 1.26	13.05 ± 3.18		0.000
CK-MB (U/L)	17.51 ± 5.75	66.31 ± 42.79		0.000
cTnT (ng/L)	7.03 ± 2.35	123.35 ± 69.67		0.000
NTpro-BNP (pg/ml)	229.65 ± 59.08	373.12 ± 249.91		0.000
hs-CRP (mg/L)	2.34 ± 0.49	10.90 ± 3.76		0.000

*Abbreviations*: BMI, Body Mass Index; WBC, white blood cell; Neu, neutrophil; AST, Aspartate aminotransferase; LDH, lactate dehydrogenase; CK, Creatine Kinase; α-HBDH, alpha-hydroxybutyric dehydrogenase; mAST, Aspartate aminotransferase mitochondrial isoenzyme; TG, TG; TC, Total Cholesterol; LDL-C, Low-Density Lipoprotein Cholesterol; HDL-C, High-Density Lipoprotein Cholesterol; Lp(a), Lipoproteins a; ApoA1, ApolipoproteinA1; ApoB100, ApolipoproteinB100; sLDL-C, small dense Low-Density Lipoprotein; NEFA, Nonestesterified Fatty Acid; Hcy, Homocysteine; CK-MB, Creatine Kinase, MB Form; cTnT, cardiac troponin T; NTpro-BNP, N-terminal pro-B type natriuretic peptide; hs-CRP, hypersensitive-CRP.

### Levels of lncRNA PELATON were increased in ACS individuals

**Figure 1:**
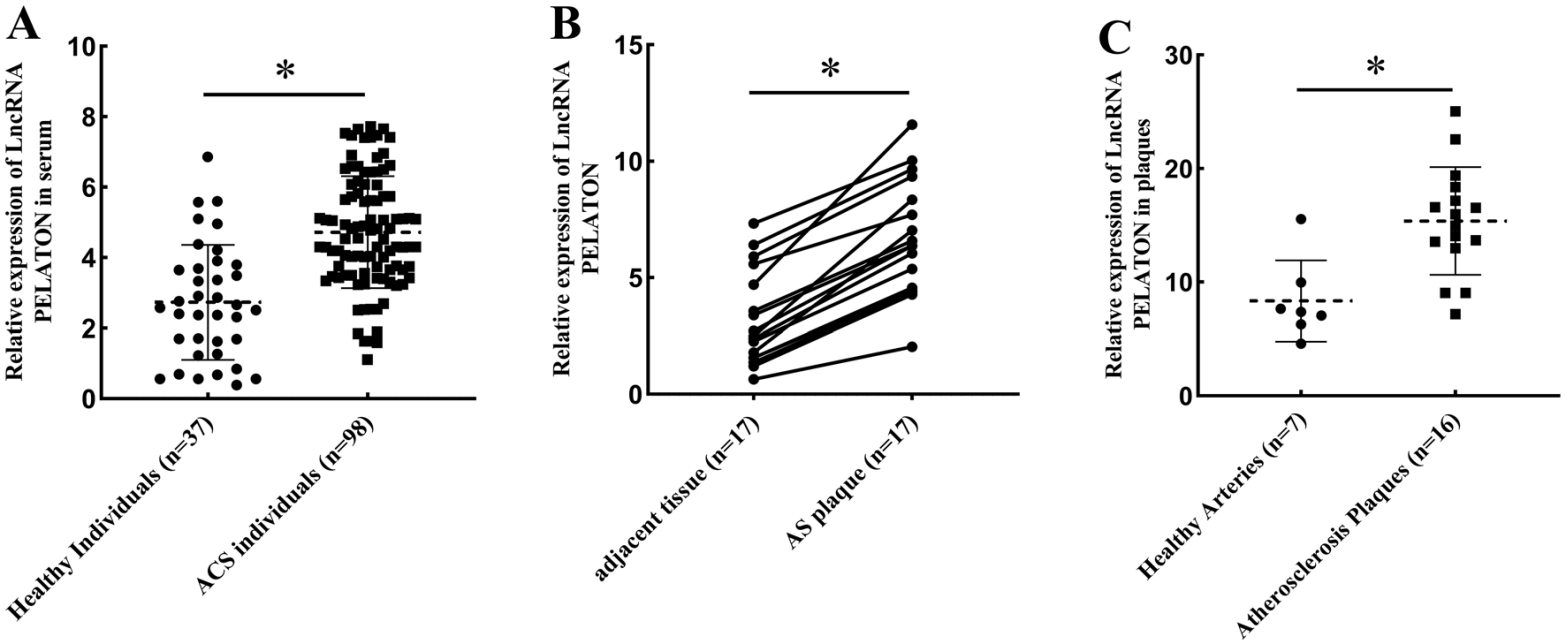
Expression of lncRNA PELATON in ACS. (**A**) Level of lncRNA PELATON in patients with ACS and healthy individuals. (**B**) Level of lncRNA PELATON in atherosclerotic plaques and adjacent tissue in the coronary artery. (**C**) Level of lncRNA PELATON in atherosclerotic plaques and healthy arteries in the carotid artery. * indicates p < 0.05.

Compared with healthy individuals, the expression of lncRNA PELATON was markedly upregulated in ACS individuals (Fig. 1A). Plaque and surrounding tissue were obtained in some patients who underwent coronary artery surgery. LncRNA expression was higher in plaques than that in adjacent intimal tissues in almost all patients (n = 17) (Fig. 1B). Atherosclerotic plaques (n = 16) and healthy arteries (n = 7) were obtained from patients who underwent carotid endarterectomy. The expression of lncRNA PELATON was increased in atherosclerotic plaques (Fig. 1C). The above experiments indicated that the expression of the lncRNA PELATON was closely related to the occurrence of plaques.

### Correlation between lncRNA PELATON expression and clinical features of ACS individuals

To investigate whether lncRNA PELATON was related to the clinical parameters, all patients with ACS were categorized into a relatively low (n = 51)/high (n = 47) level serum lncRNA PELATON group, according to the median expression of LncRNA PELATON. Detailed information about the clinical characteristics of the 98 ACS individuals is presented in Table 2, including sex, age, BMI, diabetes mellitus, hypertension, number of diseased vessels and Grace scores. These data suggested that high expression of lncRNA PELATON in serum was significantly related to age (*p* = 0.005), BMI (*p* = 0.001), hypertension (*p* < 0.001), number of diseased vessels (*p* < 0.001) and Grace scores (*p* = 0.001), indicating that high serum lncRNA PELATON expression may account for the advanced stage of ACS. However, there was no apparent relationship between increased serum lncRNA PELATON expression and gender or diabetes mellitus.

**Table 2:** Correlations between the serum LncRNA PELATON with clinical characteristics.

Characteristics	Total	Level of LncRNA PELATON		χ2 value	p
		Low, n = 51	High, n = 47		
**Gender**				2.356	0.124
Male	59	18	41		
Female	76	33	43		
**Age**				7.863	0.005
≤ 50 years	54	35	19		
> 50 years	44	16	28		
**BMI**				10.42	0.001
≤ 24	50	34	16		
> 24	48	17	31		
**Diabetes mellitus**				1.316	0.251
Yes	36	16	20		
No	62	35	27		
**Hypertension**				13.75	< 0.001
Yes	63	24	39		
No	35	27	8		
**Diseased vessels**				12.00	< 0.001
≤ 2 vessels	18	16	2		
≥ 3 vessels	80	35	45		
**Grace scores**				13.26	0.001
Low risk	14	12	2		
Middle risk	20	14	6		
High risk	64	25	39		

### Correlation of lncRNA PELATON expression in ACS individuals with hs-CRP, CK-MB, cTnT and NTpro-BNP

**Figure 2:**
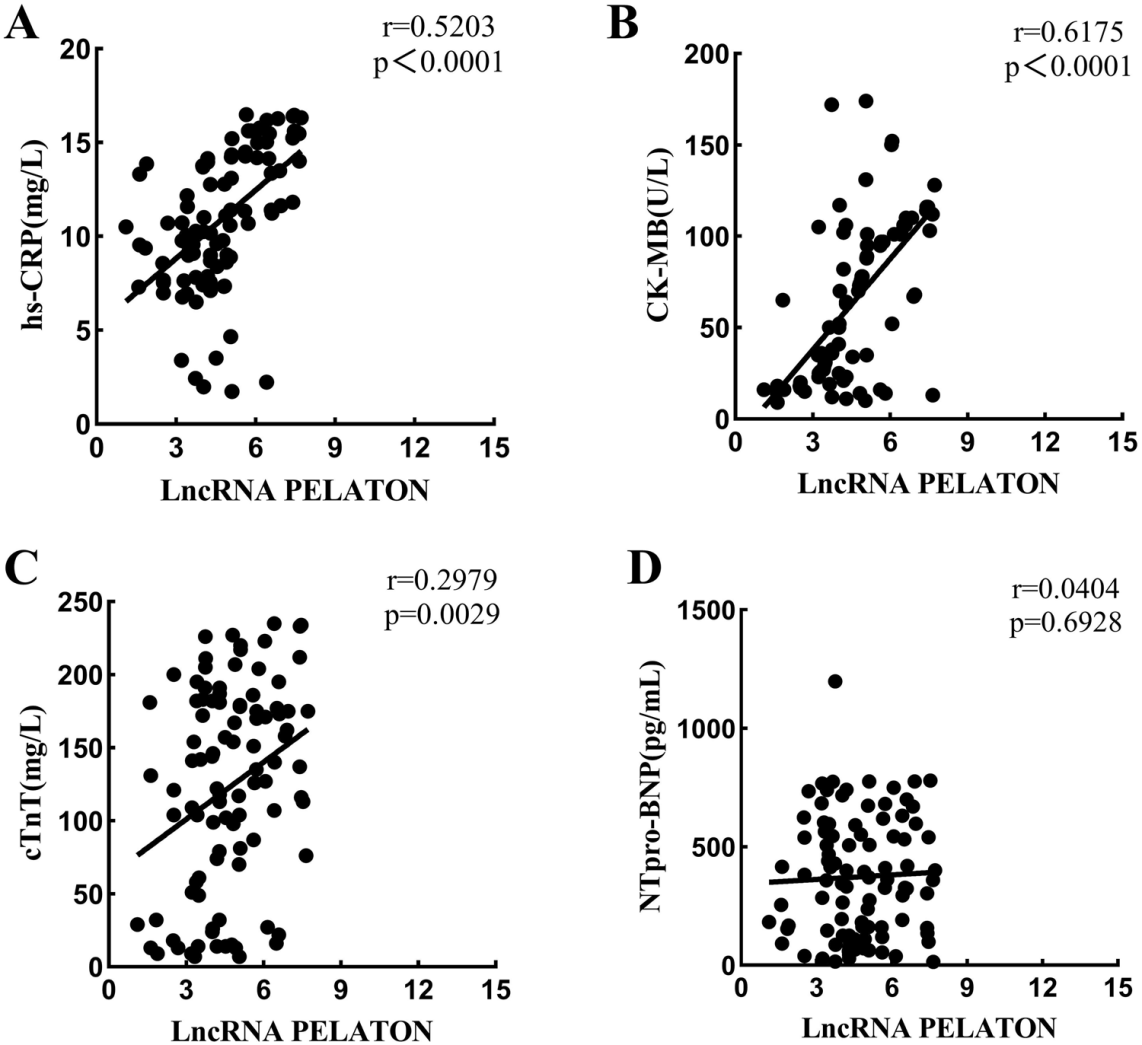
Correlation analysis of the expression of lncRNA PELATON with the concentration of myocardial indicators. (**A**) Correlation between lncRNA PELATON and hs-CRP; (**B**) Correlation between lncRNA PELATON and CK-MB; **C.** Correlation between lncRNA PELATON and cTnT; (**D**) Correlation between lncRNA PELATON and NTpro-BNP.

Pearson’s linear correlation coefficient was used to analyze the correlation of lncRNA PELATON expression in ACS individuals with hs-CRP, CK-MB, cTnT and NTpro-BNP. The results revealed that lncRNA PELATON expression was positively correlated with hs-CRP (p < 0.0001), CK-MB (p < 0.0001) and cTnT (p = 0.0029) (Fig. 2A, B and C), while no significant correlation was observed with NTpro-BNP (p = 0.6928) (Fig. 2d).

### Diagnostic value of serum lncRNA PELATON for ACS

**Figure 3:**
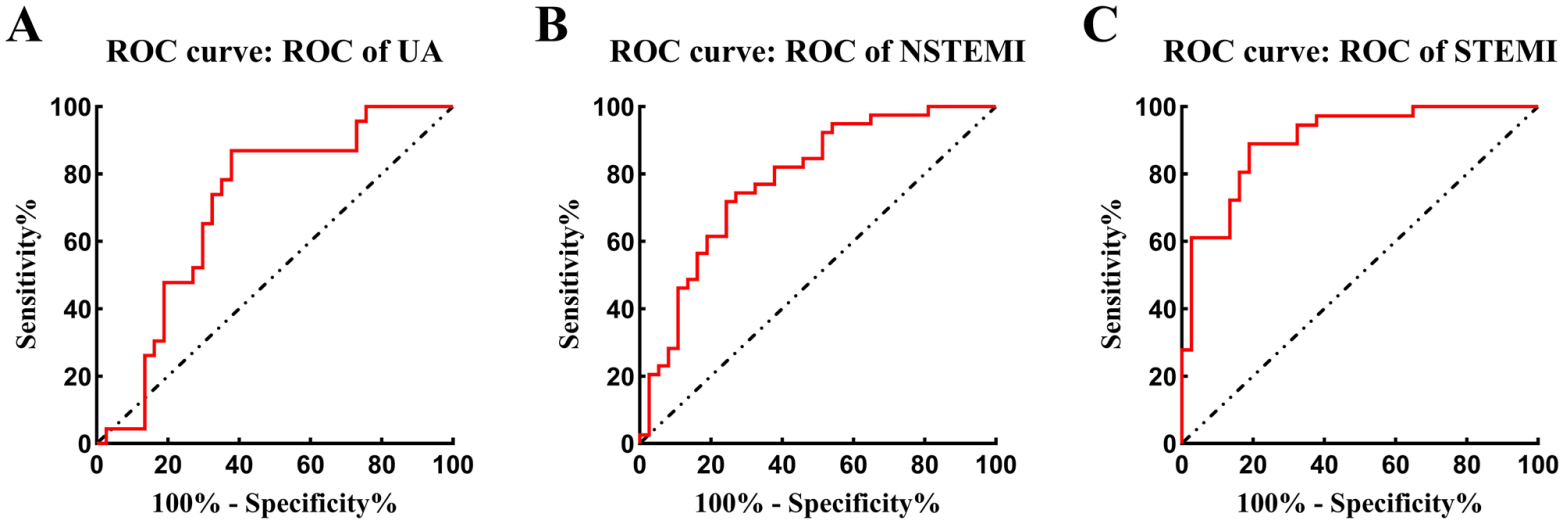
Receiver operating characteristic curve for evaluating UA, NSTEMI or STEMI by lncRNA PELATON. (**A**) ROC curve for evaluating UA; (**B**) ROC curve for evaluating NSTEMI; (**C**) ROC curve for evaluating STEMI.

We evaluated the diagnostic value of serum lncRNA PELATON levels for UA, NSTEMI or STEMI. The ROC curve results showed that the expression of lncRNA PELATON could distinguish UA individuals from healthy individuals, and the AUC was 0.7056. Moreover, the sensitivity was 86.96%, and the specificity was 62.16% (Fig. 3A and Supplementary Table 1). The ROC curve results showed that the expression of lncRNA PELATON could distinguish NSTEMI individuals from healthy individuals, and the AUC was 0.782. Moreover, the sensitivity was 71.79%, and the specificity was 75.68% (Fig. 3B and Supplementary Table 1). The ROC curve results showed that the expression of lncRNA PELATON could distinguish STEMI individuals from healthy individuals, and the AUC was 0.900. Moreover, the sensitivity was 88.90%, and the specificity was 81.10% (Fig. 3C and Supplementary Table 1).

### Combination of lncRNA PELATON and CK-MB as diagnostic biomarkers of NSTEMI or STEMI

**Figure 4:**
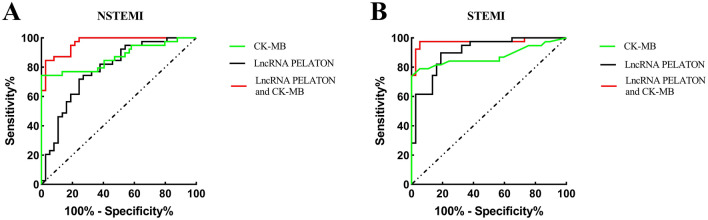
Evaluating ACS by lncRNA PELATON and CK-MB. (**A**) ROC curve for evaluating NSTEMI; (**B**) ROC curve for evaluating STEMI.

We evaluated the diagnostic value of serum lncRNA PELATON combined with CK-MB for NSTEMI or STEMI. A ROC curve (Fig. 4A) showed that the lncRNA PELATON combined with CK-MB could distinguish NSTEMI individuals from healthy individuals, and the AUC was 0.966. Moreover, the sensitivity was 84.62%, and the specificity was 97.3% (Supplementary Table 2). The lncRNA PELATON combined with CK-MB could distinguish STEMI individuals from healthy individuals, and the AUC was 0.973 (Fig. 4B). Moreover, the sensitivity was 97.44%, and the specificity was 89.19% (Supplementary Table 2).

### Survival analysis of ACS individuals

**Figure 5:**
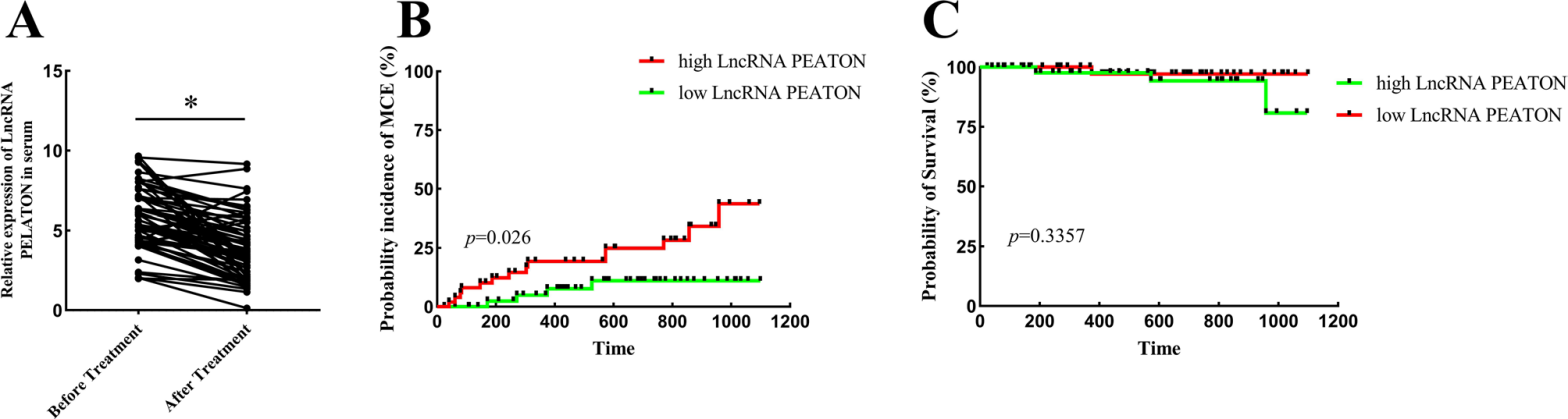
Survival analysis of ACS individuals. (**A**) The expression of lncRNA PELATON in patients with treatment. (**B**) The incidence of MCE in ACS patients between high and low lncRNA PELATON expression. (**C**) The probability of survival between high and low lncRNA PELATON.

Serum lncRNA PELATON levels were remeasured in 68 patients at 1 year after admission (1 patient died within 1 year, and 29 patients were diagnosed less than a year after their last diagnosis). The results showed that the level of serum lncRNA PELATON after treatment in patients was significantly decreased (Fig. 5A). Next, we determined whether lncRNA PELATON expression correlates with future adverse cardiovascular events (MCE). The major cardiovascular events included cardiovascular death, myocardial infarction and stroke. We found that high lncRNA PELATON expression levels in serum were associated with a higher incidence of MCE (*p* < 0.05) (Fig. 5B). However, the probability of survival between high and low lncRNA PELATON had no significant difference (*p* = 0.3357) (Fig. 5C).

## Discussion

The pathological process of AS begins when circulating low-density lipoproteins (LDL) stimulate endothelial cell damage, causing endothelial cell dysfunction, activating endothelial secretory cell adhesion factors and chemokines that promote leukocyte recruitment, and inducing inflammatory cell infiltration^26^. Increased vascular permeability and infiltration of monocytes that differentiate into macrophages promote the formation of foam cells^27^. Simultaneously, local hypoxic stimulation of the vascular intima induces angiogenesis, which extends to the vascular intima and promotes the development of AS^28^. LncRNAs can act on pathological processes at different stages, such as endothelial dysfunction, angiogenesis, and immune response, to play an important role in the process of AS ^29,30^. Based on the important role of lncRNAs in gene transcription, we believe that there are still many unknown lncRNAs waiting to be discovered. However, it is inefficient to find the association between new lncRNAs and diseases through traditional experimental methods. Fortunately, after various biological experiments and RNA sequencing, a large amount of biological data on the association between noncoding RNAs (lncRNAs, circRNAs and miRNAs) and diseases have been accumulated. Based on RNA sequencing, RNA expression profiling, disease-directed acyclic graphs, RNA-gene interactions, disease-gene associations and RNA-disease associations, Chen et al. developed several effective computer prediction models to clarify the link between RNA and disease and further verified the validity of the above models through biological methods^15,31,32^. Undoubtedly, this is one of the effective ways to elucidate the important role of noncoding RNA in the future.

There is one point worth discussing when reviewing the literature. From the literature, it has been emphasized that lncRNA PELATON is specifically expressed in monocyte-macrophage cell lines, and its expression in multiple cell lines (monocytes, macrophages, endothelial cells, fibroblasts, smooth muscle cells) has also been tested in the article^23^. Our experiment also confirmed its specific expression. However, known as RP11-290F20.3, GCRL1 (gastric cancer-related lncRNA 1)^24^ is still expressed in gastric cancer tissues, gastric cancer cell lines and nude mice. However, when we conducted bioinformatics to query the miRNA related to lncRNA PELATON, it was found that it was only expressed in human species and could not be queried in mice. Although the specificity of its cell and tissue expression does not affect its important role in ACS, this problem is still worthy of further discussion.

The incidence of important cardiovascular events is a factor affecting the prognosis of patients. Our study found that the incidence of important cardiovascular events in ACS patients with high expression of lncRNA PELATON was significantly higher than that of ACS patients with low expression of lncRNA PELATON. However, during the follow-up period, we did not observe a difference in mortality between the two groups of patients. We speculate that this may be related to the lack of follow-up time, which is different from the common 1-year, 5-year or even 10-year survival rate of cancer patients. Generally, ACS patients have a long survival time with right diagnosis and proper treatment. However, some patients have poor medication compliance in clinical practice. For follow-up experiments, we need to strengthen patient compliance education to eliminate it. At the same time, we hope to extend the follow-up time to observe the impact of lncRNA PELATON on long-term mortality and major cardiovascular events in patients with ACS.

Previous studies have shown that the lncRNA PELATON is highly expressed in AS plaque^23^. The lncRNA PELATON can enhance the phagocytic capacity of macrophages and increase the production of reactive oxygen species by increasing CD36 expression on the macrophage membrane^23^. Our study found that lncRNA PELATON was significantly increased in the serum and plaques of ACS patients, and it was aggravated with the degree of blood vessel blockage. Taking into account the pathological basis of ACS disease, lncRNA PELATON has a significant effect of promoting plaque formation, and the content of lncRNA PELATON will increase significantly when macrophages are formed into foam cells. Therefore, we speculate that lncRNA PELATON and plaque block formation promote each other. Foam cell formation and foam cell mobility are important processes of AS formation when plaques are retained^33^. LncRNA PELATON can upregulate the expression of CD36 to promote foam cell formation. The significance of this study lies in the discussion of the clinical significance of lncRNA PELATON, including its diagnostic significance for ACS disease and its influence on subsequent ACE and death of ACS disease. This method could also be implemented to reveal the function of other LncRNAs in ACS. However, the lncRNA PELATON was chosen for the experiments based on the following two points: lncRNA PELATON is specifically expressed in the monocyte-macrophage cell line, which are two important cells involved in atherosclerosis; the expression level of lncRNA PELATON is gradually increased during monocyte-macrophage-foam cell transformation, indicating that it may be closely related to the progression of atherosclerosis. We believe that the clinical importance of lncRNAs should be paid more attention first. However, the specific mechanism of lncRNA PELATON affecting AS formation was not further discussed in this paper. Compared with the current traditional experimental methods, computer models based on bioinformatics databases may be more efficient and effective in predicting lncRNA action networks (such as lncRNA-miRNA interactions). For example, Zhang et al. explored and predicted potential interactions between lncRNAs and miRNAs based on a semisupervised interaction group network, demonstrating superiority and good generalization^34^. Additionally, a network distance analysis model for lncRNA-miRNA association prediction (NDALMA) obtained satisfactory results in fivefold cross-validation. All this work will be used in combination with cell and animal experiments in our next study^35^.

In conclusion, our experiments demonstrated that higher levels of lncRNA PELATON were negatively correlated with the prognosis of ACS, which could be used as a potential index to assess the incidence and prognosis of ACS.

## Materials and methods

### Subjects

The ACS individuals diagnosed at the Sun Yat-sen University affiliated Zhongshan Hospital from July 2018 to September 2021 were enrolled retrospectively. This was in line with the following inclusion criteria: (1) patients diagnosed with ACS for the first time and (2) patients satisfying the diagnostic criteria of ACS in accordance with the Diagnostic and Therapeutic Criteria of the Cardiovascular system and diagnosed by coronary angiography. Exclusion criteria: (1) patients with hepatic and renal insufficiency; (2) patients allergic to iodine or contrast agents; (3) patients with severe cardiopulmonary insufficiency; (4) patients with uncontrolled severe arrhythmias; (5) patients with severe electrolyte disorder; and (6) patients with severe infection. Serum samples and laboratory results from 37 healthy patients during the same period were collected. All patients agreed to participate in the experiment and signed an informed consent form. These study results had no influence on the subsequent management of patients. This study was conducted in accordance with the Declaration of Helsinki and was approved by the Ethics Board of the Sun Yat-sen University affiliated Zhongshan Hospital.

### Clinical data collection and follow-up

We obtained the clinical characteristics and laboratory test results of all enrolled subjects through our hospital’s computerized test system (Nanfang Huiqiao). Patients were followed from July 2018 to September 2021. The follow-up information of patients was obtained through systematic case data in our hospital and telephone conversations.

### Real-Time Polymerase Chain Reaction (RT–PCR)

Total RNA was isolated from human tissue, serum and cell lines using the miRNEasy kit (Qiagen). It was reverse transcribed using the TaqMan microRNA reverse Transcription Kit (Thermo Fisher, Waltham, MA, USA). Next, RT–PCR was performed using 2 × SYBR Green PCR Master Mix. Glyceraldehyde 3-phosphate dehydrogenase (GAPDH) was the internal reference. LncRNA PELATON: Forward: 5’-CCTTCCTTCTGCCTCCACTG-3’ and Reverse: 5’-CCTTCCT TCTGCCTCCACTG-3’, and GAPDH: Forward: 5’-GCTCTCTGCTCCTCCTGTTC-3’ and Reverse: 5’-ACGACCAAATCCGTTGACTC-3’.

### Statistical methods

GraphPad 8.0 and SPSS 26.0 software were used for statistical analysis of the data. The chi-square test was used for nonparametric data, and the mean ± standard deviation (SD) was used for descriptive continuous variables. The independent samples or paired samples t test was used for data analysis if the data followed a normal distribution. If the data did not obey the normal distribution, the Mann–Whitney U test was used for data analysis. The Kaplan–Meier method was used for the analysis of survival curves, and the log-rank method was used for inspection. The flowchart of the whole analysis process is shown in Supplementary Fig. 1. A *p value* < 0.05 indicated that the difference was statistically significant.

## Supplementary Information

Supplementary Information.

## Data Availability

All data generated or analyzed during this study are included in this published article (and its Supplementary Information files).
