# Supplementary material for: High expression of lncRNA PELATON serves as a risk factor for the incidence and prognosis of acute coronary syndrome
Source: Sci Rep. 2022 May 16;12:8030. doi: 10.1038/s41598-022-11260-2 (PMC9110396; doi:10.1038/s41598-022-11260-2)

**Supplement materials**

Table 1 Evaluating ACS by LncRNA PELATON

|  | AUC | p | Sensitivity (%) | Specificity (%) | Youden index |
| --- | --- | --- | --- | --- | --- |
| LncRNA PELATON for UA | 0.706 | 0.007 | 86.96% | 62.16% | 0.491 |
| LncRNA PELATON for NSTEMI | 0.782 | ＜0.0001 | 71.79% | 75.68% | 0.474 |
| LncRNA PELATON for STEMI | 0.900 | ＜0.0001 | 88.90% | 81.10% | 0.699 |

Table 2 Evaluating ACS by LncRNA PELATON and CK-MB

|  | AUC | p | Sensitivity (%) | Specificity (%) | Youden index |
| --- | --- | --- | --- | --- | --- |
| LncRNA PELATON and  CK-MB for NSTEMI | 0.966 | ＜0.0001 | 84.62% | 97.3% | 0.819 |
| LncRNA PELATON and  CK-MB for STEMI | 0.973 | ＜0.0001 | 97.44% | 89.19% | 0.866 |

Figure1 The flowchart of the whole experiment process


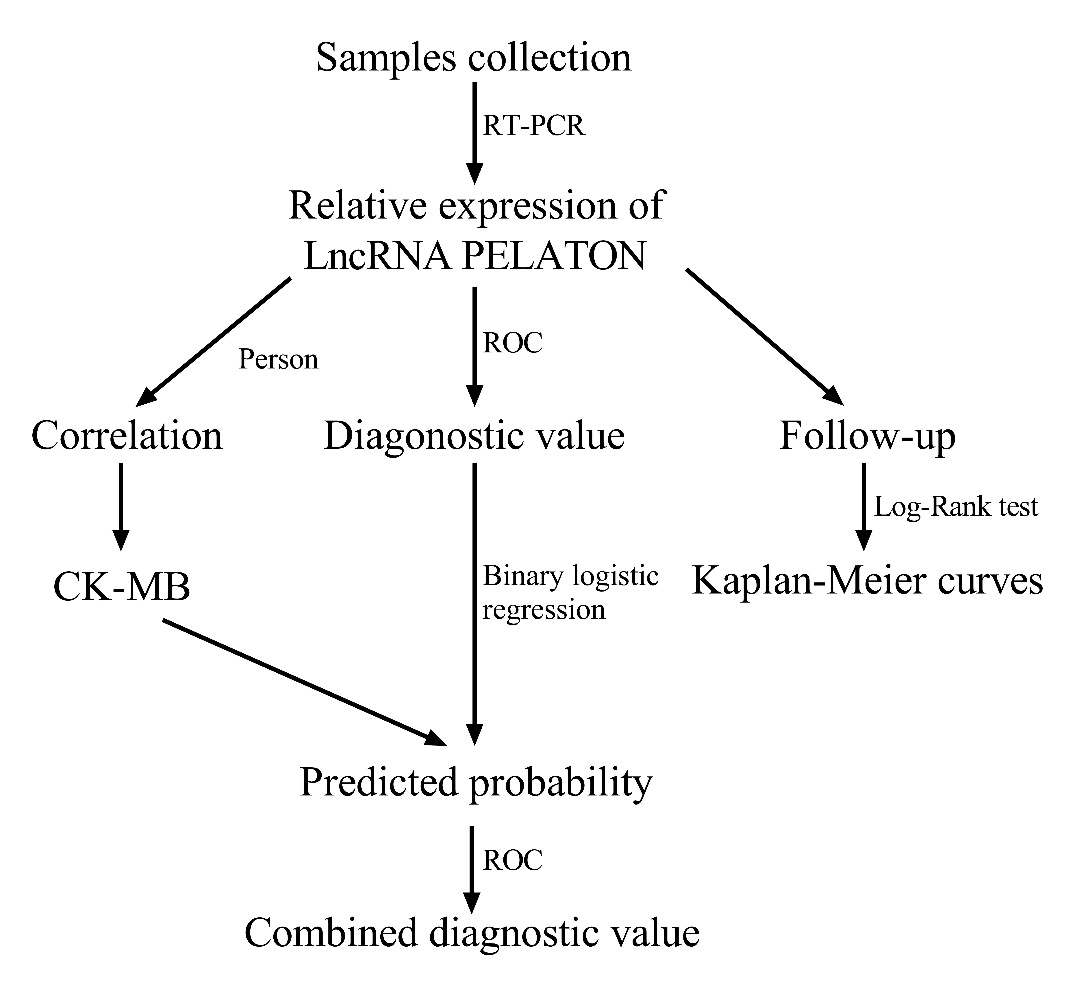

Supplement: Supplementary file 1 — Supplementary Information. [file 41598_2022_11260_MOESM1_ESM.docx]
